# The value of health and well-being from a societal perspective: A willingness to pay experiment in the Netherlands

**DOI:** 10.1007/s10198-025-01814-2

**Published:** 2025-07-29

**Authors:** Karen Trujillo Jara, Daphne C. Voormolen, Werner Brouwer, Job van Exel

**Affiliations:** 1 Erasmus School of Health Policy & Management (ESHPM), Erasmus University Rotterdam, Burgemeester Oudlaan 50, 3062 PA Rotterdam, The Netherlands; 2 Erasmus Centre for Health Economics Rotterdam (EsCHER), Erasmus University Rotterdam, Rotterdam, The Netherlands

**Keywords:** Health, Well-being, Threshold, Contingent valuation, Societal perspective

## Abstract

Interpreting the results of cost-effectiveness analyses requires a threshold value for the costs per quality-adjusted life year (QALY). The first empirical studies that estimated this threshold value from a societal perspective in the Netherlands were conducted 10 years ago. This paper is aimed at estimating the social willingness to pay (WTP) per QALY and per well-being adjusted life year (WALY); investigating how the societal value of a QALY changed over the course of about ten years and comparing the WTP for well-being relative to health from a societal perspective. In this study, the contingent valuation approach was used, in which QALYs and WALYs were valued under uncertainty and corrected for probability weighting. The estimates obtained in a representative sample of the Dutch population ranged from €27,800 to €95,300 per QALY, depending on the specification of the societal perspective. The value of health found in the SOC and SII versions of this study, nominally, were between 6.1% and 33.4% higher than the values found 10 years ago. Moreover, the estimates per WALY ranged from €88,500 to €349,500. Finally, our results show that a year in full well-being was valued between 2.95 and 4.35 times higher than a year in full health.

## Introduction

Economic evaluations can inform allocation decisions in health care by providing evidence about the (incremental) costs and benefits of alternative health interventions. In conventional economic evaluations, relevant outcomes are typically confined to *health* benefits captured in terms of quality-adjusted life-years (QALYs), which comprise both length and health-related quality of life (HRQoL) [1, 2]. HRQoL is commonly measured using multi-attribute utility instruments like the EQ-5D or the SF6D, which allow computing a utility score for all possible health states measured by these instruments on a scale that is anchored on the state dead (with value 0) and the state of full or perfect health (with value 1) [3, 4]. Subsequently, the ratio of incremental costs and health effects (ICER) can be calculated [5] and compared to a relevant threshold, which, depending on the perspective chosen for the analysis (and assumed aim of the healthcare decision maker), may reflect the cost-effectiveness of displaced healthcare or the societal willingness to pay (WTP) for health gains [6–8]. This threshold assists healthcare decision makers in making (optimal) allocation decisions.

In very general terms, the decision rule then is

1 $$Tq\Delta -\Delta c>0$$

in which *T*_*q*_ denotes the appropriate threshold value (i.e., conventionally health opportunity costs or consumption value of the QALY), ΔQ denotes the change in the relevant outcome (typically QALYs) and $\Delta c$ denotes the change in relevant costs. Equation (1) can be rewritten as

2 $$\frac{\Delta c}{\Delta Q}<{T}_{q}$$

which highlights that the decision rule implies that incremental costs per incremental unit of relevant outcome should not exceed the value of a unit of that outcome. In other words, the ICER of an intervention should remain below the threshold value. This emphasizes two things which are central in the current paper. First, within a chosen perspective, it is crucial to have a sound conceptualization and estimation of the threshold because without an appropriate threshold, interventions ultimately cannot be evaluated as being health or welfare improving. Secondly, when using other outcome measures than health-related QALYs, like measures of well-being, as is increasingly discussed also within healthcare, threshold values that are relevant for those outcomes need to be derived as they may not be, nor are expected to be, equal to the value of a QALY. Both topics will be highlighted further below.

The importance of sound estimates of thresholds for economic evaluations using QALYs as outcome measure is increasingly being recognized. First estimates of opportunity costs within the healthcare sector (i.e., cost-effectiveness of displaced care), relevant when adopting a healthcare perspective or when adopting a societal perspective but operating under a non-optimal budget [8–10] have been reported. For the Netherlands, for instance, investigating the marginal returns of spending on cardiovascular disease (CVD) hospital care resulted in an estimate of €41,000 per QALY gained [10]. In the US, a threshold of US$104,000 per QALY was reported [11], while the average figures for Spain and England were reported to be €25,000 and £12,936 per QALY, respectively [12, 13]. These estimates thus differ substantially between countries (and/or applied methods and contexts).

When adopting a societal perspective, the societal consumption value of marginal health gains, often expressed as the monetary value of health, should inform the relevant threshold. Estimates of this value have also been reported. Empirical studies in the field of healthcare typically use stated preferences (such as WTP techniques or choice experiments) methods to estimate the consumption value of a QALY, both from an individual (valuing own health gains) and a broader societal perspective (valuing own and/or others’ health gains) [14]. Most studies apply an individual perspective. For Germany, a WTP per QALY was estimated at US$16,285 [15]; for Denmark, US$17,685 [16]; for Sweden, US$32,253 [17]; and for Malaysia, US$65,464 [18]. Also here, empirical estimates differ substantially between countries. A recent systematic review showed a predicted mean empirical value of US$52,619 per QALY from an individual perspective [14].

Empirical research in estimating a monetary value of a QALY using a societal perspective are more scarce [19]. Arguably, such estimates are, however, relevant in the context of collectively funded healthcare systems such as in the Netherlands. Then, the threshold does not only reflect individual valuations but also societal considerations, for instance regarding equity [20–23]. Currently, in the Netherlands, a threshold is used that increases with severity of illness, based on societal notions of equity and solidarity, in general ranging from €20,000 for treatments of mild diseases to €80,000 per QALY for treatments of severe diseases. The first study that reported a societal value of a QALY was conducted ten years ago by Bobinac et al. [20], using a comprehensive design, valuing health gains under uncertainty, while correcting for probability weighting. The estimated results averaged between €26,200 and €81,500 per QALY, depending on assumptions regarding probability weighting and whether the person him- or herself could (social-inclusive-individual; SII) or could not (Social value; SOC) also be a beneficiary of the treatment [24]. In addition, a recent study in Japan estimated a societal WTP per QALY in a range between US$27,300 and 58,300 [25]. In the current study, we will replicate the study of Bobinac et al. [20], to investigate the current societal value of a QALY. This also allows us to investigate how this value has changed over the past 10 years. Moreover, we will apply the same method to estimate the value of a year in full well-being using a broader outcome measure, the ICEpop CAPability measure for Adults (ICECAP-A) [26]. Because both values are estimated in the same population, it allows us to directly compare the societal values of health and well-being.

The comparison of the monetary value of a QALY with that of a broader outcome measure like the ICECAP-A is relevant since such broader outcome measures are increasingly receiving attention. Not only in terms of discussion about their relevance (next to or replacing QALYs) but also in terms of development and validation of other well-being instruments like the ICECAP-O [27], the Well-being of Older People (WOOP) instrument [28], the Health and Well-being (EQ-HWB) instrument [29], and the 10-item Well-being instrument (WiX) [30]. These outcome instruments attempt to measure (different conceptualizations of) well-being or general quality of life, from the notion that health and social care interventions can produce benefits beyond health [31]. If these benefits are not properly measured and valued in economic evaluations, interventions may not be appropriately assessed, potentially leading to suboptimal decisions. This may especially (but not exclusively) be the case for interventions in long-term care, social care, and palliative care [32–34]. The use of these instruments in actual economic evaluations is expected to increase, also since some national guidelines offer more room for other outcome measures than QALYs [35]. However, this then also requires having sound estimates of the monetary value of well-being. Empirical estimates of the monetary value of well-being gains are, however, still very scarce. Himmler et al. [49], using the well-being method and the ICECAP-A measure of capability well-being, provided a first estimate for a year in full well-being of £66,597 in a UK sample, while using the same method and sample, the value of a QALY was estimated to be £30,786. Brinkmann et al. [36], using the ICECAP-A, a short WTP format and a large European sample, find a range of values for a year in full well-being, ranging from €13,323 for larger gains and €61,375 for smaller gains. To our knowledge, no estimates of the value of a year in full well-being from a societal perspective have been reported so far.

Hence, the current study has two aims. First, we wanted to replicate Bobinac et al. [20] to investigate how the societal value of a QALY changed over the course of ten years. Such replication studies are scarce, and to our knowledge this is the first of its kind in this field. General inflation, growth in wealth and life expectancy, and the COVID-19 pandemic, among others, may have impacted the value attached to health gains. By using the same methodology and study design as Bobinac et al. [20], it is possible to compare the results between these two studies directly, and to compute the growth rate in the value of a QALY over this 10 year period, which can inform the discount rate for health effects in countries applying a differential discount rate for costs and effects, like the Netherlands [37–39]. To our knowledge, this is the first study that can calculate a growth rate in this way. Secondly, by using a very similar study design for investigating the value of a well-being adjusted life-year (WALY) as for a QALY, this study aims to provide a first estimate of the value of well-being from a societal perspective, valuing these gains under uncertainty and adopting both a social-inclusive-individual perspective (SII; respondent may be beneficiary next to others) as well as a purely social value (SOC; respondent is not a beneficiary). This contributes to the scarce literature in this area, and to the determination of thresholds when using outcome measures broader than QALYs in economic evaluations. In addition, by using the same study population, a direct comparison can be made between the social value of a QALY and a WALY (using the EQ-5D and ICECAP-A instruments, respectively), which is an additional novelty of this study.

## Methods

This study replicated the approach used by Bobinac et al. [20]. From September 26th to October 25th 2022, a sample of 1,013 respondents was recruited by the sampling agency Dynata, quota-sampled to be representative for the adult population of the Netherlands (age 18–65 years), gender and education level.

### Questionnaire structure

The introduction to the questionnaire included warm-up questions regarding the willingness to pay for a pair of shoes and a car, and a graphical illustration for respondents to get familiar with the concept of risk and to improve their understanding of the exercises (e.g., Corso et al. [40]; Donaldson et al. [41]; see Appendix A). Next, the questionnaire to elicit values for a QALY and a WALY was structured as follows:

- Respondents were randomized to one of two versions of the questionnaire, one eliciting the'social-inclusive-individual value'(SII), representing the value of an expected QALY/WALY gain in others *or* the individual themselves (Version A), and another estimating the'social value'(SOC), representing the value of an expected QALY/WALY gain achieved in others (Version B) [42].
- Each version of the questionnaire consisted of two parts, one eliciting the value of health and one eliciting the value of well-being. All respondents answered the questions for part 1 “health” and for part 2 “well-being” consecutively, and within the same perspective (SII or SOC).

### Scenarios approach

This study replicates the design developed by Bobinac et al. [20]. Within the ‘SII’ and ‘SOC’ versions of part 1 “health”, respondents were randomized to one of 29 scenarios that differed in the size of the health gain that could be achieved (Appendix B). These 29 scenarios were based on 42 different health states described using the EQ-5D-3L [43] and the risk of becoming ill (i.e., 2%, 4%, 10% or 50% for the risk group).

Subsequently, for part 2 “well-being”, respondents were again randomized to one of 29 scenarios that differed in the size of the well-being gain that could be achieved (Appendix C). For this purpose, the design of the experiment for valuing a QALY was slightly adapted given that the instrument used to ask respondents to value a well-being gain has four response levels (rather than the three of the EQ-5D-3L). More specifically, the odd scenarios from the original design were kept the same while the even scenarios were scaled one level up to have all the response levels of the ICECAP-A represented in the design for valuing a WALY. In addition, the order of the levels was inverted, as shown in Appendix C (e.g., health state 1 in scenario two is 33,232 and well-being state for the same scenario is 11,212), because for EQ-5D-3L higher scores indicate *poorer* health-related quality of life while for ICECAP-A higher scores indicate *better* capability well-being. This approach resulted in comparable distributions of the marginal gains to be valued across the 29 scenarios (i.e., 0.016 for health gains and 0.017 for well-being gains).

Utilities for health states were computed using the Dutch tariff for EQ-5D-3L [44] and the risk of becoming ill, while utilities for well-being states were computed using the Dutch tariffs for the ICECAP-A [45]. At the time of designing this study, ICECAP-A was the only validated well-being instrument for which utility scores were available.

### Data collection

A total of 1,013 respondents completed the online questionnaire, 511 the ‘SOC’ version and 502 the ‘SII’ version. In parts 1 and 2 of the questionnaire, respondents were first asked to indicate which of the two (health/well-being) states was better and which was worse, and then asked to rate them using a visual analog scale (VAS). Respondents were subsequently presented with a hypothetical scenario (see Appendix E) in which a certain group of the population currently was in the (health/well-being) state they indicated to be the best but was at risk of getting into the (health/well-being) state they indicated to be the worse for a period of one year due to infection with a virus. In addition, they were informed that the risk and non-risk groups were identical in all other respects.

The risk of getting sick was completely avoidable by taking a medicine (without side effects) every month during that year. This medicine could be financed from an increase in the monthly premium for health insurance for all adult people in the Netherlands. In the ‘SOC’ version, the respondent did not belong to the risk group, while in the ‘SII’ version he or she did. Respondents were asked to indicate their willingness-to-pay (WTP) for this treatment that would eliminate the risk of the (health/well-being) deterioration. The payment mechanism was explained to respondents as an increase in the monthly health insurance premium, which every adult in the Netherlands would be required to pay as taking health insurance is compulsory. The WTP was elicited in a two-step approach. First, using a payment scale, respondents initially reported the highest amount they would certainly pay for the treatment, and the lowest amount they would certainly not pay on the same scale. Next, respondents provided a point estimate of their maximum WTP using an ‘open-ended’ (OE) format, bounded by the WTP values obtained in the first step. If maximum WTP was 0 respondents were asked for a motivation.^1^

The questionnaire was pilot tested for feasibility in a random sample of 100 respondents.

### Analysis

For the analysis, the total number of expected QALYs (TEQ) and WALYs (TEW) in each scenario was calculated as follows:

3 $$TEQ=U\left(HS1\right)-U\left(HS2\right)*p*\left(\mathrm{13,500,000}*0.5\right)$$

4 $$TEW=U\left(WS1\right)-U\left(WS2\right)*p*\left(\mathrm{13,500,000}*0.5\right)$$

where U(HS1)-U(HS2) concerns the quality-of-life decrement and U(WS1)-U(WS2) the well-being decrement in the scenario presented to the respondent, and *p* corresponds the probability of the specific decrement occurring in half of the adult population of the Netherlands^2^ (i.e., 13,500,000 * 0.5) presented to the respondent. Because individuals tend to overweight small and underweight large probabilities, as prospect theory suggests [46], and might treat probabilities non-linearly, similar to Bobinac et al. [20], the analysis were conducted with probabilities uncorrected (NW) and corrected for probability weighting using two functional forms (Tversky & Kahneman [46]; Prelec [47]) and the parameters elicited by Bleichrodt & Pinto [48] (see Appendix D). In addition, extremely high WTP values (i.e., highest 1%) were excluded from the analysis.

The average WTP per QALY and WTP per WALY were calculated as:

5 $$WTP per QALY=\left(<\frac{WTP\left(OEq\right)}{TEQ}>\right)*12*\mathrm{13,500,000}$$

6 $$WTP per WALY=\left(<\frac{WTP\left(OEw\right)}{TEW}>\right)*12*\mathrm{13,500,000}$$

where < WTP(OEq)/TEQ > in Eq. (4) concerns the average of the ratios between the open-ended WTP estimate and the total expected QALY gain (see (2)) for every data row, and < WTP(OEw)/TEW > in Eq. (5) the average of the ratios between the open-ended WTP estimate and the total expected WALY gain (see (3)) for every data row. These values were multiplied by 12 (i.e., the one-year period respondents would be expected to pay the increase in the monthly premium) and by 13,500,000 (i.e., the estimated number of individuals eligible to pay health insurance premiums in the Netherlands).

Sensitivity to scale was analyzed by testing whether SII and SOC estimates were affected by the size of the QALY or WALY gain on offer, using log-linear regressions. The distribution of the dependent and independent variables of interest were analyzed to determine the appropriate functional form they could adopt (e.g., the continuous variables “WTP”, “QALY gains” and “WALY gains” showed a skewed distribution and were log-transformed). Overall, ‘Log of QALY gains’, ‘age’, ‘gender’, ‘education’ and ‘income category’ were included as independent variables into the model. The variable ‘income’ was categorized from five levels (see Table 1) into three levels given the low number of observations in the extreme income brackets (46 observations in the lowest category and 89 observations in the highest). The analyses used were corrected for extremely high WTP values (by excluding the highest 1%) and for probability weighting.

**Table 1:** Summary statistics of respondents

Variables		Sample (N = 1013)
Sex	Male	48.5%
	Female	51.5%
Age	18–24	13.7%
	25–34	18.8%
	35–44	24.1%
	45–54	20.6%
	55–65	22.8%
Education	Low	16.2%
	Middle	45.1%
	High	38.7%
Income per month	Up till €999	4.5%
	€1000—€1999	17.7%
	€2000—€3499	33.6%
	€3500—€4999	23.1%
	€5000 or more	8.8%
	Prefer not to tell	12.3%
Marital status	Married	45.8%
	Living with someone	17.9%
	Single (never been married)	26.8%
	Divorced	7.0%
	Widow	0.6%
	Prefer not to tell	2.0%
Children (% yes)	1 child	40.0%
	2 children	43.6%
	3 children	11.1%
	4 children or more	5.8%
WTP for a car		20,141 (mean)
WTP for a pair of shoes		143 (mean)
Mean ICECAP-A (Dutch tariff)		0.89
Mean EQ-5D (Dutch tariff)		0.88
Mean EQ-VAS		77.2

Education level: Low (No education, Primary education, Lower vocational), Medium (Middle vocational) and High (Higher vocational, University); Income: this variable was categorized in three levels ‘Low’, ‘Medium´’ and ‘High’ for analysis purposes, and for those respondents who did not report their income ‘prefer not to tell’ a model to input the income values was conducted; ICECAP-A, ICEpop CAPability measure for Adults; EQ-5D, EuroQol-five dimensions; EQ-VAS, EuroQol-Visual Analogue Scale; WTP, willingness to pay

Finally, with the aim to handle missing values of respondents who did not report their income level (125 observations) we re-analysed the data of the results on WTP per income subgroup by imputing the income category.^3^

All analysis were conducted using STATA version 18.0.

### Ethics

This study was approved by the Research Ethics Review Committee of Erasmus School of Health Policy & Management (ETH2122-0696).

## Results

Table 1 provides an overview of the socio-economic and demographic characteristics of the sample and levels of health-related quality of life and well-being. On average, respondents completed the questionnaire in 23.4 min. The sample was representative of the general population in terms of gender and age but underrepresented in terms of low education. The warm-up questions revealed that, on average, respondents expressed a WTP of €143 for a pair of shoes, which was 28.5% lower than the value reported by Bobinac et al. [20], and a WTP of €20,141 for a car, which was 23.3% higher than the value reported in that same study.

### Descriptive analysis

Because the raw WTP values elicited from respondents are associated with different sizes of health and well-being gains presented to them (i.e., from the scenario they were randomized to), these values were rescaled to the average health and well-being gains in the design. As shown int Table 2, the mean of the WTP value for health gains in the SII version was higher than in the SOC version (i.e., €81.57 and €51.31, respectively). Similarly, the WTP value for well-being gains was higher in the SII version than in the SOC version (i.e., €423.31 and €332.86, respectively). Moreover, in both versions, the mean WTP for well-being gains was higher than the mean WTP for health gains.

**Table 2:** Descriptive statistics: WTP values rescaled to the WTP for the average health gains and well-being gains ‘SII’ & ‘SOC’

Rescaled WTP	N	Mean	SD	Median	Min	Max	[95% conf. interval]
WTP for Health gains ‘SII’	502	82	227.41	29.66	0	3,820.60	61.63 101.51
WTP for Health gains ‘SOC’	511	51	94.84	22.11	0	955.15	43.07 59.55
WTP for Well-Being gains ‘SII’	502	423	1,570.48	51.22	0	16,882.06	285.51 560.94
WTP for Well-Being gains ‘SOC’	511	333	1,372.32	48.36	0	23,517.07	213.59 452.12

### Social value of a QALY

The average health gain valued by respondents was 0.016 QALY, varying between 0.002 and 0.066 QALY. As shown in Table 3, WTP per QALY values were higher in the SII version than in the SOC version for non-weighted and weighted probabilities (ranging from €35,800 to €95,300 versus €27,800 to €65,100). In the subgroup analysis, individuals who reported a lower income (i.e., < €1,999) also reported lower WTP in the SOC version. The pattern is less clear in the SII version. Ninety-four respondents had a WTP of €0 (i.e., 40 from SII and 54 from SOC, respectively). These results were similar after imputing missing values for income. Specifically, respondents with lower income in the SOC version reported lower WTP (see Appendix F). Additionally, the WTP pattern in the SII version also remained similar, with slight variations observed in the WTP for the TK weighted values.

**Table 3:** Open-ended SOC and SII WTP per QALY estimates, weighted and non-weighted, by certainty level and income group (€, rounded to hundreds)

				SOC				SII	
		n	WTP per QALY OE NW (SD)	WTP per QALY OE TK (SD)	WTP per QALY OE P (SD)	n	WTP per QALY OE NW (SD)	WTP per QALY OE TK (SD)	WTP per QALY OE P (SD)
Mean		504/505	65,100 (94,700)	36,500 (62,700)	27,800 (54,400)	496	95,300 (165,600)	49,100 (84,600)	35,800 (63,500)
Subgroup analysis									
Certainty level	Surely not	22/23*	54,400 (95,500)	52,700* (106,100)	44,100* (91,100)	21/20*	168,700 (392,100)	45,000* (106,600)	25,800* (50,900)
	Pretty sure not	54/52*	63,500 (106,200)	23,500* (34,700)	16,700 (27,800)	40	81,800 (140,900)	43,000 (96,900)	25,600 (49,800)
	Maybe yes, maybe no	143/144*	59,300 (91,800)	32,400 (54,300)	24,300* (43,700)	150/151*	82,500 (144,900)	45,000* (77,500)	32,500* (55,700)
	Pretty sure yes	182/184*	68,600 (93,400)	41,100* (68,900)	31,400* (61,300)	162	111,500 (172,200)	57,300 (91,500)	41,900 (67,500)
	Totally sure yes	103/102*	70,200 (95,700)	37,100* (60,100)	28,400* (54,000)	123	81,400 (115,100)	46,200 (75,600)	36,900 (72,300)
Income Group	< €1,000	25	61,500 (108,200)	25,300 (37,100)	15,700 (19,600)	21	59,100 (79,800)	34,900 (75,000)	39,400** (86,800)
	€1,000—€1,999	90/91*	52,268 (74,300)	34,100* (70,200)	26,600* (62,200)	86/87*	56,000 (69,600)	57,300* (222,300)	24,900* (50,700)
	€2,000—€3,499	162/159*	75,500 (101,000)	36,200 (53,300)	27,700* (49,400)	175	113,800 (202,200)	58,400 (103,800)	40,800 (72,800)
	≥ €3,500	157/160*	71,700 (99,000)	46,500* (76,600)	36,000* (64,900)	159/158*	112,100 (181,200)	52,100* (73,300)	38,300** (59.500)

Base case data (excluding outliers 1%)

*Number of observations TK & P, if different from Open Ended (OE) responses

NW, non-weighted probabilities applied; TK, Tversky and Kahneman probability function applied (1992); P, Prelec probability function applied (1998); SOC, social value; SII, social-inclusive-individual value; QALY, quality-adjusted life year; SE, standard error

As shown in Table 4, the elicited WTP values were not sensitive to the size of the QALY gain in SII and SOC, both for weighted and non-weighted risk probabilities. WTP per QALY, however, decreased with age, was lower for ‘middle education’ in SII, both non-weighted and weighted, and was higher for the highest income group in both SOC and SII.

**Table 4:** Log-linear regressions – Health

	SII									SOC								
	NW			TK			P			NW			TK			P		
	Coeff	SE	*p*	Coeff	SE	*p*	Coeff	SE	*p*	Coeff	SE	*p*	Coeff	SE	*p*	Coeff	SE	*p*
Log (QALY gain)	−0,03	0,08	0,73	0,05	0,10	0,60	0,08	0,08	0,34	0,08	0,07	0,24	0,02	0,09	0,80	0,00	0,07	0,95
Age	−0,01	0,00	0,01	−0,01	0,00	0,02	−0,01	0,00	0,02	−0,02	0,00	0,00	−0,02	0,00	0,00	−0,02	0,00	0,00
Gender (female)	−0,02	0,11	0,85	−0,06	0,11	0,57	−0,06	0,11	0,57	−0,04	0,10	0,64	−0,05	0,10	0,63	−0,05	0,10	0,62
Education category (Low—baseline)																		
Middle	−0,42	0,15	0,01	−0,38	0,16	0,02	−0,38	0,16	0,02	−0,18	0,14	0,21	−0,17	0,14	0,24	−0,17	0,14	0,23
High	−0,32	0,16	0,04	−0,29	0,16	0,07	−0,30	0,16	0,07	−0,23	0,14	0,11	−0,20	0,14	0,17	−0,20	0,14	0,17
Income category (Low—baseline)																		
Middle	0,20	0,14	0,16	0,19	0,14	0,18	0,20	0,14	0,17	0,42	0,12	0,00	0,36	0,13	0,00	0,37	0,13	0,00
High	0,50	0,14	0,00	0,45	0,15	0,00	0,45	0,15	0,00	0,36	0,12	0,00	0,37	0,13	0,00	0,37	0,13	0,00
Constant	3,55	0,42	0,00	3,87	0,45	0,00	3,94	0,38	0,00	4,18	0,37	0,00	3,83	0,39	0,00	3,76	0,33	0,00
	N = 404 Adj R2 = 0.0460			N = 404 Adj R2 = 0.0332			N = 404 Adj R2 = 0.0348			N = 392 Adj R2 = 0.1062			N = 393 Adj R2 = 0.0795			N = 393 Adj R2 = 0.0958		

Dependent variable: log (WTP)

NW, non-weighted probabilities applied; TK, Tversky and Kahneman probability function applied (1992); P, Prelec probability function applied (1998); SOC, social value; SII, social-inclusive-individual value; QALY, quality-adjusted life year; SE, standard error

The value of health found in the SOC and SII versions of this study, nominally, were between 6.1% and 33.4% higher than the values found 10 years ago by Bobinac et al. [20]. The implicit growth rate for the value of health is, therefore, 0.6% to 2.9% per year.

### Social value of a WALY

The average well-being gain valued by respondents was 0.017 WALY, varying between 0.0001 and 0. 185 WALY. Similar to WTP per QALY values, the values for a WALY in the SII version were higher than in the SOC version for non-weighted and weighted probabilities, ranging from €105,500 to €349,500 versus €88,500 to €283,400 (see Table 5). In the subgroup analysis, respondents who were (relatively) certain about their answers reported higher WTP, while respondents in the lowest income group (i.e., < €1,999) reported lower WTP for a WALY in the SOC version and the highest WTP per WALY in the SII version. Ninety-five respondents had a WTP of €0 (i.e., 45 from the SII and 50 from SOC, respectively). After imputing missing values for the income category, WTP values decrease for categories low and middle in SII format and increase for the same income categories in SOC (see Appendix F).

**Table 5:** Open-ended SOC and SII WTP per WALY estimates, weighted and non-weighted, by certainty level and income group (€, rounded to hundreds)

				SOC				SII	
		n	WTP per WALY OE NW (SD)	WTP per WALY OE TK (SD)	WTP per WALY OE P (SD)	n	WTP per WALY OE NW (SD)	WTP per WALY OE TK (SD)	WTP per WALY OE P (SD)
Mean		505	283,400 (662,200)	128,400 (282,200)	88,500 (193,600)	495	349,500 (955,300)	155,400 (394,600)	105,500 (259,900)
Subgroup analysis									
Certainty level	Surely not	25	127,900 (298,500)	59,400 (121,800)	41,800 (80,700)	24	193,800 (396,500)	95,800 (196,600)	69,700 (149,700)
	Pretty sure not	50	210,400 (449,500)	104,700 (230,300)	77,300 (190,600)	35	103,200 (160,500)	43,900 (62,900)	28,700 (39,200)
	Maybe yes, maybe no	142	249,500 (699,400)	111,200 (304,400)	75,800 (209,300)	166	389,500 (1,057,000)	172,200 (430,900)	116,600 (277,400)
	Pretty sure yes	186	322,400 (645,200)	144,000 (268,400)	98,300 (179,000)	152	342,600 (10,500)	159,800 (446,000)	112,800 (299,200)
	Totally sure yes	102	333,400 (780,000)	152,500 (322,500)	105,300 (216,400)	118	406,700 (880,700)	171,400 (353,700)	110,500 (232,400)
Income Group	< €1,000	24	227,600 (570,000)	94,300 (228,200)	60,500 (143,400)	20	573,900 (1,285,913)	260,800 (546,100)	180,900 (367,100)
	€1,000—€1,999	89	209,000 (540,158)	99,100 (270,600)	70,700 (207,800)	88	217,900 (497,400)	93,200 (197,800)	60,600 (126,000)
	€2,000—€3,499	162	307,100 (660,000)	136,800 (268,800)	93,000 (176,400)	175	371,300 (970,800)	164,200 (395,800)	111,100 (259,300)
	≥ €3,500	160	340,400 (695,200)	158,300 (298,600)	111,300 (208,500)	157	336,700 (950,100)	154,100 (403,100)	106,800 (272,200)

Base case data (excluding outliers 1%)

NW, non-weighted probabilities applied; TK, Tversky and Kahneman probability function applied (1992); P, Prelec probability function applied (1998); SOC, social value; SII, social-inclusive-individual value; WALY, well-being adjusted life year; SD, standard deviation

The elicited WTP for well-being shows sensitivity to the size of WALY gains both in the SII and SOC versions (see Table 6), whether non-weighted or weighted (i.e., coefficients range between 0.08 and 0.10). Furthermore, age and income were significantly associated with the WTP for well-being.

**Table 6:** Log-linear regressions – Well-being

	SII									SOC								
	NW			TK			P			NW			TK			P		
	Coeff	SE	*p*	Coeff	SE	*p*	Coeff	SE	*p*	Coeff	SE	*p*	Coeff	SE	*p*	Coeff	SE	*p*
Log (WALY gain)	0,08	0,03	0,03	0,09	0,04	0,03	0,09	0,04	0,04	0,10	0,03	0,00	0,09	0,04	0,01	0,08	0,04	0,05
Age	−0,01	0,00	0,00	−0,01	0,00	0,00	−0,01	0,00	0,00	−0,02	0,00	0,00	−0,02	0,00	0,00	−0,02	0,00	0,00
Gender (female)	−0,02	0,10	0,86	−0,02	0,10	0,83	−0,02	0,10	0,83	−0,12	0,10	0,24	−0,12	0,10	0,25	−0,12	0,10	0,26
Education category (Low—baseline)																		
Middle	−0,28	0,15	0,06	−0,28	0,15	0,06	−0,28	0,15	0,06	−0,14	0,15	0,35	−0,14	0,15	0,34	−0,15	0,15	0,32
High	−0,31	0,15	0,04	−0,31	0,15	0,04	−0,31	0,15	0,04	−0,33	0,15	0,03	−0,34	0,15	0,03	−0,34	0,15	0,03
Income category (Low—baseline)																		
Middle	0,30	0,14	0,03	0,30	0,14	0,03	0,30	0,14	0,03	0,45	0,13	0,00	0,46	0,13	0,00	0,46	0,13	0,00
High	0,62	0,14	0,00	0,62	0,14	0,00	0,62	0,14	0,00	0,60	0,13	000	0,60	0,13	0,00	0,61	0,14	0,00
Constant	3,92	0,29	0,00	3,95	0,30	0,00	3,90	0,29	0,00	4,12	0,29	0,00	4,02	0,29	0,00	3,90	0,28	0,00
	N = 398 Adj R^2^ = 0.0806			N = 398 Adj R^2^ = 0.0807			N = 398 Adj R^2^ = 0.0790			N = 395 Adj R^2^ = 0.1317			N = 395 Adj R^2^ = 0.1246			N = 395 Adj R^2^ = 0.1194		

Dependent variable: log (WTP)

NW, non-weighted probabilities applied; TK, Tversky and Kahneman probability function applied (1992); P, Prelec probability function applied (1998); SOC, social value; SII, social-inclusive-individual value; WALY, well-being adjusted life year; SE, standard error

## Discussion

Empirical studies aimed at estimating a monetary value of a QALY using a societal perspective remain scarce. Nonetheless, these estimates are relevant, particularly in the context of interpreting the outcomes of economic evaluations for decision making in collectively funded healthcare systems, based on notions of (income and risk) solidarity, such as that in the Netherlands. Moreover, little is known empirically about the growth rate of the value of health. The latter is interesting in general and directly relevant for countries using differential discounting of costs and benefits [38, 39]. In addition, given that health and social care interventions can have benefits beyond health, broader outcomes measures that capture general quality of life, or well-being, receive increasing attention in recent years. In order to interpret the results of economic evaluations in which such outcome measures are used, the monetary value of well-being gains needs more investigation. Both QALY and WALY values were derived in this study under risk and using both a socially inclusive (SII) and purely other regarding (SOC) perspective. As Bobinac et al. [20] indicated: “*The ex ante SII insurance perspective may be considered most relevant in estimating the willingness to pay for health gains in a collectively funded system. […] The SII perspective combines the individual’s self-interest with the concern for others and aligns well with the context of the social insurance system.*” Obviously, in applying (the results of) a SII, double-counting individual welfares needs to be avoided [49]. Moreover, there may be situations in which the purely other regarding (SOC) estimates may be more relevant (e.g., when considering funding diseases that respondents or premium payers cannot experience themselves).

In this study, we therefore replicated the study of Bobinac et al. [20] to investigate the current societal value of a QALY and its growth rate during the past decade. Moreover, we expanded this study to estimate the value of a WALY, using a similar methodology, study design and population, allowing for direct comparisons between the values of health and well-being. Using a sample of 1,013 respondents from the Netherlands, we estimated a WTP per QALY ranging from €27,800 to €65,100 in the SOC version and from €35,800 to €95,300 in the SII version. These values were between 6.1% to 33.4% higher than the values reported by Bobinac et al. [20] ten years ago, implying an annual growth rate of between 0.6% and 2.9%. The WTP per WALY values, as expected based on well-being representing a broader outcome than health, were higher than those for QALYs, and ranged from €88,500 to €283,400 and from €105,500 to €349,500 in the SOC and SII versions, respectively. The elicited WTP values for QALYs and WALYs suggest that WALY gains are between 2.95 and 4.35 more valuable than health gains.

Our WTP per QALY estimates are in line with the reference values currently used in the Netherlands [50, 51], and a study using an individual approach to valuing health gains under risk (e.g. Bobinac et al. [52]). They are higher than those presented in Bobinac et al. [53], but in that study changes were valued under certainty. Interestingly, the estimates presented here also reasonably align with Dutch estimates of threshold values estimating the marginal cost-effectiveness of current healthcare spending. A study estimating this value considering hospital care spending in the Netherlands resulted in an estimated (‘supply-side’) threshold of €73,600 per QALY, with intervals ranging from €53,000 to €94,000 per QALY (Stadhouders et al., 2019). In addition, Van Baal et al. [10], focusing on CVD healthcare spending, reported an estimate of €41,000 per QALY gained.

Comparing our results to previous estimates of WTP per QALY is hampered by the fact that other studies used different designs, samples and methods. Our results regarding the WTP per WALY are higher than the estimates reported in the study of Brinkmann et al. [36], where the average WTP per WALY for the Netherlands ranged from €14,613 to €58,952. Note that the average well-being gains valued in their more explorative and as part of a larger questionnaire less controlled study, using a different design, were substantially larger than the well-being gains valued in our study. The latter estimate in that sense is most comparable to our estimates, although even that refers to larger (less marginal) well-being gains compared to this study, which may partly explain the difference especially in light of lack of sensitivity to scale. The study by Himmler et al. [54], again using a different design, i.e. estimating the value of capability well-being in the UK using the well-being valuation approach, reported an average value of a WALY of £66,597 – slightly lower than the lower estimates in our range. That study suggested that the value of a WALY was between 1.7 and 2.6 times larger than that of a QALY, which is a lower relative value as compared to the results presented here.

Before highlighting some implications of our findings, several limitations of our study need to be emphasized.

First, like in the original study, we used an online questionnaire which may have affected the quality of the data acquired. This is compounded by the fact that the valuation tasks presented to the respondents were complex. For instance, imagining being in one health (or well-being) state with the risk of dropping to a worse state, typically both hypothetical, is not easy. Whether respondents were able to fully understand and imagine what it would mean to be in these states while also abstracting from their own current health (or well-being) requires further investigation, ideally also using qualitative studies enabling a better understanding of observed responses. Moreover, whether they fully adhered to the described scenarios (e.g. did not imagine they could affect the exogenous risk of moving to the worse state other than through the offered treatment) also could be investigated further in future studies (for instance using think-out-load designs).

Second, the fact that our SII estimates were higher than the SOC estimates, as well as the fact that our WALY estimates were higher than our QALY estimates is encouraging in terms of our methods and respondents being sensitive to *scope* of what is being valued. However, the results for sensitivity to *scale* were less consistent. The regressions appear to signal theoretical validity for the WALY estimates, but not for the QALY estimates as the elicited WTP for QALY gains were not significantly associated with the size of the QALY gain on offer. While this is similar to the original study, it is a clear limitation. As Bobinac et al. [20] pointed out, this may be related to the fact that people in such a context may have broader considerations when eliciting WTP (e.g. paying for having a publicly funded health care system in general – e.g. Hurley, [55] – or WTP as an expression of the ‘act of giving’ – e.g. Kahneman and Knetsch [56]; Andreoni [57]; Olsen [58]). However, the contrast with WTP for WALYs is interesting and difficult to explain in this context. For the WTP per WALY estimates, even with theoretical validity, ‘theoretical plausibility’ of the sensitivity to scale, i.e. whether the increase in WTP is sufficiently proportional to the increase in size of the gain, is also important [36, 59].

Third, we only focused on the Netherlands and, hence, cannot generalize our results to other countries. Focused and elaborate international studies, that also allow international comparisons of the results (e.g. Brinkmann et al. [36]), are recommended. Moreover, our study sample was not fully representative of the general population in the Netherlands in terms of education (with an underrepresentation of low educated respondents). Likewise, we focused on two specific instruments to measure health and well-being (i.e., EQ-5D and ICECAP-A). Generalizing to other instruments requires attention. For well-being, the ICECAP was the only well-being instrument for which Dutch utility tariffs were available at the time of the study. Given the development of new well-being instruments (e.g., the Health and Well-being (EQ-HWB) instrument [29] and the 10-item Well-being instrument (WiX) [30]) and in absence of one preferred well-being instrument, replications of studies like the one presented here with these new instruments are encouraged. In addition, while we used tariffs for the EQ-5D-3L that were country specific, we used somewhat older tariffs (i.e., those reported by Lamers et al. [44]). If preferences in the Dutch general public for health states changed over time, this may have affected our findings.

Fourth, some design choices deserve emphasis. We, for instance, adjusted our results for probability weighting but did so using existing weighting functions rather than individually elicited values. Also, we valued avoiding a health loss rather than obtaining a health gain, which likely affected our estimates. Moreover, we used a payment scale in this experiment, which may have affected the elicited answers (also through the range of values on the scale; Soeteman et al. [60]). Next to this, our payment vehicle was an increase in monthly health insurance premium. While this is the common way of financing healthcare interventions in the Netherlands, where everyone is obliged to purchase basic health insurance and, therefore, familiar with such a premium as payment, also this choice may have affected our results and could affect comparability with studies using other payment vehicles. It needs emphasis that using premium as payment vehicle for respondents may have been more recognizable and common for health than for well-being, which may have influenced our results. Similarly, public knowledge and discussions about the height of and increases in *health*care premiums may have influenced the marginal willingness to pay for health relative to the marginal willingness to pay for well-being. These aspects should be investigated further. In addition, while it is common to value marginal changes in willingness to pay studies, this is not always reflective of real decisions in healthcare. Then, often it is known with certainty *who* is affected and the gains and losses in health or well-being often are non-marginal. Therefore, translating our findings to that decision-making context requires attention as well. The same holds for the fact that we valued health and well-being changes, not mortality changes. Finally, individual responses to willingness-to-pay experiments can be aggregated in different ways to obtain a mean value of a QALY or WALY (i.e., mean of ratios or ratio of means), which likely affects results [16]. Here, we followed the same approach as the original study and computed the mean of ratios.

Finally, it needs acknowledging that our intention of strictly replicating the original study of Bobinac et al. [20] in order to preserve comparability, also required some choices that may be considered suboptimal from other viewpoints. This, for instance, pertains to the age range chosen (i.e., 18 to 65) while the retirement age in the Netherlands changed between the original and current study, the fact that respondents only answered one WTP scenario [20], as well as the use of the EQ-5D-3L while nowadays the EQ-5D-5L may be considered the preferred instrument. This also means that the implications from this study need to be drawn in that context. Moreover, in the description of the scenarios in the original and, therefore, also the current study, respondents were asked to imagine that the potential impacts on health and well-being were caused by ‘a virus’. Given the fact that between the original and current study the COVID pandemic took place, we cannot rule out that this description may have influenced our results as the description may be considered ‘less neutral’ in the current than in the original study. This holds for health effects as well as well-being effects. In that sense, it would be interesting to investigate whether other assumed causes of the health and well-being effects would lead to other results – and whether any potential effect of the proximity in time to the COVID-19 pandemic would diminish over time [36].

Some implications of this study, while acknowledging the limitations above, are that the current estimates of the value of a QALY align reasonably well with the range of values currently used in the Dutch decision-making context (€20,000 to €80,000 per QALY). It should be noted that this range is related to the severity of illness in the population under consideration (e.g. Reckers-Droog et al. [21]), which is not the case in our estimates. Furthermore, given the similar methodology and design of our study and that from 2013, we could also infer growth rates in the value of health, which is a unique feature of this study and one that is directly relevant for economic evaluations in the Netherlands. The estimated annual growth rates of 0.6 to 2.9% have also informed the new guideline for economic evaluation in healthcare in the Netherlands [61], where an average growth rate of 1.5% was chosen to allow discount rates of 3% for costs and 1.5% for effects. This, therefore, implicitly corrects *future* thresholds to account for the growth in the value of health. For other countries using a societal perspective it is also important to know that the value of health may grow over time, either in the context of discounting or in the context of updating thresholds. Regarding the latter, it may also be important to reconsider the threshold values used in the Netherlands, or even adjusting them on a yearly basis. In absence of a better estimate, the above-mentioned growth rate could be used for this purpose. That would also ensure consistency with the discounting practice. This would make it even more important to periodically re-estimate (the growth rate in) the value of health and well-being. Moreover, it may also be worthwhile to investigate whether the thresholds per equity class all change at the same rate.

Our results for the value of a WALY (unsurprisingly) suggest that this broader outcome has a higher value than a QALY. This implies that when using broader outcome measures in economic evaluations, which is sometimes required given the (intended or expected) outcomes of an intervention beyond health, also requires using different thresholds. Our estimates are somewhat higher than previous estimates in the literature and thresholds would ideally be informed by more studies in this area. This highlights the need for more research and clear guidance for interpreting economic evaluations using broader outcome measures, either next to or instead of QALYs.

In conclusion, this study provides new estimates of the value of a QALY, using a similar methodology and study design as done 10 years ago [20]. The results show that the value of health has grown over time, with an estimated growth rate of 0.6–2.9% per year, which has implications for (future) thresholds and differential discounting in health economic evaluations. Moreover, our study highlights that the value of a WALY is higher than that of a QALY, but more research is required in this emerging area as well as better guidelines for incorporating broader outcomes in healthcare decision-making.

## Figures and Tables

**Table 7:**
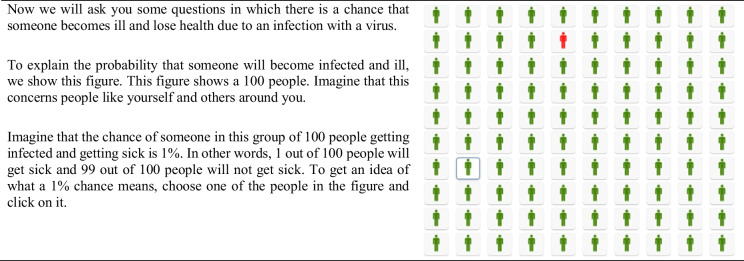
Concept of risk, The concept of risk was shown to respondents as follows (translated from Dutch; based on Donaldson et al., 2010):

## Data Availability

Study data is available upon request from the corresponding author.

## Appendix A

Table 7

## Appendix B

Table 8

**Table 8:** Design of scenarios, levels of risk and qaly gain

Choice scenario	Health state 1	Health state 2	Level of uncertainty (%)	Expected QALY gain
1	22222	11131	10	0.02
2	33232	33323	50	0.009
3	21312	12111	2	0.007
4	22323	21312	2	0.007
5	22323	12111	2	0.015
6	21232	32211	4	0.01
7	11112	22121	10	0.008
8	11122	22122	10	0.012
9	21323	22233	4	0.012
10	22331	21133	4	0.007
11	21111	12121	50	0.066
12	23232	32232	50	0.028
13	11312	11113	10	0.014
14	12311	11211	2	0.007
15	32311	12311	10	0.016
16	32311	11211	2	0.01
17	21111	12211	50	0.039
18	32313	32331	50	0.002
19	11211	22211	4	0.005
20	23313	11133	50	0.042
21	11121	22112	10	0.016
22	12223	13332	10	0.014
23	11312	11211	2	0.008
24	11332	11312	4	0.013
25	11332	11211	2	0.014
26	21222	33321	2	0.008
27	22222	13311	50	0.042
28	11112	22112	4	0.005
29	33212	32223	4	0.009

## Appendix C

Table 9

**Table 9:** Design of scenarios, levels of risk and waly gain

Choice scenario	Well-being state 1	Well-being state 2	Level of uncertainty (%)	Expected WALY gain
1	33333	44424	10	0.0021
2	11212	11121	50	0.0705
3	34243	43444	2	0.002
4	22121	23132	2	0.0049
5	33232	43444	2	0.0046
6	23212	12233	4	0.0003
7	44443	33434	10	0.0034
8	33322	22322	10	0.0184
9	34232	33322	4	0.0001
10	22113	23311	4	0.0026
11	34444	43434	50	0.0106
12	21212	12212	50	0.0062
13	44243	44442	10	0.0005
14	32133	33233	2	0.0038
15	23244	43244	10	0.011
16	12133	33233	2	0.008
17	34444	43344	50	0.0103
18	12131	12113	50	0.0415
19	44344	33344	4	0.0015
20	21131	33311	50	0.1846
21	44434	33443	10	0.0043
22	32221	31112	10	0.0188
23	44243	44344	2	0.0019
24	33112	33132	4	0.0047
25	44223	44344	2	0.0031
26	23222	11123	2	0.0067
27	33333	42244	50	0.0574
28	33332	22332	4	0.0074
29	22343	23332	4	0.0007

## Appendix D

Table 10

**Table 10:** Non-weighted and weighted probabilities

Non-weighted probabilities (%)	Weighted probabilities (%)	
	Functional form (Tversky and Kahneman, 1992):	Functional form (Prelec, 1998):
	Parameter estimate (Bleichrodt and Pinto, 2000): for losses	Parameter estimate (Bleichrodt and Pinto, 2000):
50	45	44
10	17	21
4	10	16
2	6	13

From “Valuing QALY gains by applying a societal perspective,” Bobinac et al., 2013, Journal of Health economics, 22, p. 1276

## Appendix E. Example of the willingness-to-pay question

The introduction to the question was as follows:

Suppose that all adult Dutch people are currently in the (health state/well-being state) that you rated as the best state in the previous question. Half of these Dutch adults are at risk of becoming ill tomorrow and end up in a worse health state due to some virus. We call this the risk group. The other half of the adult Dutch population is not at risk. Furthermore, these groups are completely similar (in terms of age, gender, lifestyle, etc.)

Imagine that you belong to the risk group and therefore are at risk of becoming ill due to the virus [in SII format]/Imagine that you do not belong to the risk group and therefore are not at risk of becoming ill due to infection with the virus [in SOC format]. Anyone who becomes ill will remain in the worse (health state/well-being state) for one year. After that, their (health/well-being) improves back to the health condition you rated as the best

In the risk group [in SOC format], including you [in SII format], the chance of getting into the worse (health/well-being) condition is 2% (4%/10%/50%). This means that 2 (4/10/50) in 100 people end up in the poor (health/well-being) status and (100–2/100–4/100–10/100–50) in 100 people remain in the better (health/well-being) status. The figure on the right shows the chance that people in the risk group have of becoming ill

The risk of falling into the poor (health/well-being) condition is completely avoidable by taking a drug every month (without side effects). Everyone (all 100 people) will then certainly remain in the best (health/well-being) state

The government wants to protect the risk group against the chance of becoming ill. The drug is financed by an increase in the monthly premium for health insurance for all Dutch adults. So, the people who are not at risk also pay through their premium. Everyone pays the premium from their own (family) income. After 12 months, the monthly premium will return to its current level

The WTP elicitation proceeded as follows:

Respondents were asked to indicate how much they would be willing to pay for the drug that would reduce the risk of the health/well-being deterioration to zero, through an increase in their monthly health insurance premium

- First, they were asked to indicate on a payment scale the highest amount they would certainly pay and subsequently the lowest amount they would certainly not pay for this medicine. [Ordered low-to-high payment scale with monthly installments of (€) 0, 1, 2, 3, 4, 5, 6, 8, 10, 12, 15, 18, 20, 22, 25, 30, 40,50, 75, 100, 150, 250, 500].
- Next, the respondents were asked to indicate in an ‘open-ended’ format the exact WTP for this medicine, within the range they specified on the payment scale.
- Finally, they were asked, ‘Please indicate how sure they were about your WTP answer.’ [Answer options were as follows: (i) totally sure yes; (ii) pretty sure yes; (iii) maybe yes, maybe no; (iv) pretty sure not;(v) surely not.

## Appendix F

Table 11

**Table 11:** Open-ended SOC and SII WTP per QALY and WALY estimates, weighted and non-weighted, by income group§ (€, rounded to hundreds)

				SII				SOC	
Subgroup analysis		n	WTP per QALY OE NW (SD)	WTP per QALY OE TK (SD)	WTP per QALY OE P (SD)	n	WTP per QALY OE NW (SD)	WTP per QALY OE TK (SD)	WTP per QALY OE P (SD)
**Income Group**	<€1000	21	59.100 (79.800)	43.200 (84.200)	39.400 (86.800)	25	61.500 (108.300)	25.300 (37.100)	15.700 (19.600)
	€1000 - €1999	109/110*	61.700 (79.500)	36.300* (71.600)	26.100* (49.800)	122/123*	49.500 (75.800)	30.300* (62.400)	23.200* (54.500)
	€2000 - €3499	205	104.500 (189.900)	54.800 (98.300)	39.000* (70.200)	199/196*	70.200 (99.400)	33.900* (51.500)	25.800* (47.100)
	≥ €3500	161/160*	111.000 (180.300)	51.500* (73.0000)	38.000* (59.200)	158/161*	71.300 (98.900)	46.200* (76.400)	35767.53* (64.700)
		**n **	**WTP per WALY OE NW (SD) **	**WTP per WALY OE TK (SD) **	**WTP per WALY OE P (SD) **	**n **	**WTP per WALY OE NW (SD) **	**WTP per WALY OE TK (SD) **	**WTP per WALY OE P (SD) **
**Income Group**	<€1000	20	573.900 (1.285.913)	260.800 (546.100)	180.900 (367.100)	24	227.600 (570.000)	94.300 (228.200)	60.500 (143.400)
	€1000 - €1999	111	233.200 (489.800)	99.500 (194.500)	64.800 (123.500)	121	204.100 (501.800)	93.900 (245.400)	65.400 (185.800)
	€2000 - €3499	205	403.500 (1.104.600)	177.600 (448.600)	119.900 (290.500)	199	292.000 (728.600)	129.500 (295.000)	87.800 (190.800)
	≥ €3500	159	332.800 (944.700)	152.600 (400.800)	105.800 (270.600)	161	340.700 (693.000)	158.100 297.700	87.800 (190.800)

§observations imputed by income

## Footnotes

1 Respondents could indicate: (i) I am unable to pay more than €0; (ii) I don't think it's worth more than €0; (iii) I do not want to pay because of ethical concerns; and (iv) other. All zero responses were included in the analysis.

2 Based on number provided by the Centraal Bureau voor de Statistiek (www.cbs.nl).

3 The imputation model used data on gender, age, education category and (part time or full time) employment.
